# Dihydroartemisinin Induces Growth Arrest and Overcomes Dexamethasone Resistance in Multiple Myeloma

**DOI:** 10.3389/fonc.2020.00767

**Published:** 2020-05-15

**Authors:** Ying Chen, Rui Li, Yuqi Zhu, Sixia Zhong, Jinjun Qian, Dongqing Yang, Artur Jurczyszyn, Meral Beksac, Chunyan Gu, Ye Yang

**Affiliations:** ^1^The Third Affiliated Hospital of Nanjing University of Chinese Medicine, Nanjing, China; ^2^School of Medicine & Holistic Integrative Medicine, Nanjing University of Chinese Medicine, Nanjing, China; ^3^Department of Internal Medicine, University of Iowa, Iowa City, IA, United States; ^4^Department of Hematology, Collegium Medicum, Jagiellonian University, Kraków, Poland; ^5^Department of Hematology, School of Medicine, Ankara University, Ankara, Turkey; ^6^Key Laboratory for Combination of Acupuncture and Chinese Materia Medica of Chinese Ministry of Education, Nanjing University of Chinese Medicine, Nanjing, China

**Keywords:** dihydroartemisinin, artemisinin, multiple myeloma, dexamethasone, drug resistance

## Abstract

The discovery of artemisinin (ART) for malaria treatment won the 2015 Nobel Prize in Medicine, which inspired the rediscovery and development of ART for the treatment of other diseases including cancer. In this study, we investigated the potential therapeutic effect of ART and dihydroartemisinin (DHA) on multiple myeloma (MM) cells including primary MM cells and in 5TMM3VT mouse model. Both *in vitro* and *in vivo* experiments showed that DHA might be a more promising anti-MM agent with significantly improved efficacy compared to ART. Mechanistic analyses suggested that DHA activated the mitochondrial apoptotic pathway by interacting with ferrous (Fe^2+^) ions and oxygen to produce reactive oxygen species (ROS). Intriguingly, DHA could reverse the upregulated expression of B-cell lymphoma 2 (Bcl-2) protein, a typical mitochondrial apoptotic marker, induced by dexamethasone (Dexa) in MM. We further demonstrated that DHA treatment could overcome Dexa resistance and enhance Dexa efficacy in MM. Additionally, DHA combined with Dexa resulted in increased ROS production and cytochrome C translocation from the mitochondria to the cytoplasm, resulting in alterations to the mitochondrial membrane potential and caspase-mediated apoptosis. In summary, our study demonstrated that DHA was superior to ART in MM treatment and overcame Dexa resistance both *in vitro* and *in vivo*, providing a promising therapeutic strategy for MM therapy.

## Introduction

Multiple myeloma (MM) remains an incurable hematological malignancy of plasma cells, despite the therapeutic advances over the past two decades with numerous agents including proteasome inhibitors such as bortezomib (1), ixazomib (2), and carfilzomib (3), monoclonal antibodies such as elotuzumab (4), and daratumumab (5), immunomodulatory drugs such as pomalidomide (6) and lenalidomide (7), and other treatments including chimeric antigen receptors (CAR)-T-cell therapy (8, 9) gaining clinical approval. Dexamethasone (Dexa) remains the most widely used drug for the treatment of MM despite the development of resistance in patients after prolonged exposure to its high doses (10), which in turn, is associated with poor prognosis of MM. Recurrent MM patients treated with numerous anti-MM drugs or high-risk patients with MM have poor median survival rates (11); thus, novel molecular targeting therapies are required to overcome drug resistance in MM treatment.

An increasing number of studies have demonstrated that artemisinin (ART)-type based endoperoxide drugs exhibit anticancer properties (12, 13). Dihydroartemisinin (DHA)-inhibited proliferation of cancer cells may be associated with the production of reactive oxygen species (ROS) (14, 15), induction of apoptosis (16), inhibition of angiogenesis (17), and ferroptosis (18). For example, in lung cancer, DHA-inhibited proliferation induces cell cycle arrest and decreases tumor growth by suppressing invasion and migration, increases the concentration of calcium (Ca^2+^) ions, and activates p38 (19). In glioma, DHA induces autophagy, apoptosis, and cell cycle arrest by increasing the cleavage of caspase-3, decreasing the expression of protein kinase B (p-AKT), and downregulating AKT phosphorylation followed by caspase-3 activation (20). Furthermore, artemisinin-type drugs increase the sensitivity of resistant cancer cells to conventional drugs (21). Examples of this include DHA combined with gemcitabine, which is efficacious against pancreatic tumor cells by suppressing gemcitabine-induced nuclear factor kappa-light-chain-enhancer of activated B cells (NF-κB) activation (22); DHA combined with cyclophosphamide, which inhibits spontaneous pulmonary metastasis (23), and a combination of DHA and gemcitabine, which decreases hepatoma tumor growth (24). However, the synergistic effects of DHA with the conventional drug Dexa for the treatment of MM have not yet been determined.

In the present study, two artemisinin-type drugs, ART and DHA, were examined for their anti-MM effect *in vitro* and *in vivo*. We also established their mechanism of action.

## Materials and Methods

### Cell Culture

Human MM cell lines MM.1S, MM.1R cells were purchased from ATCC (CRL-2974 and CRL-2975, respectively), ARP1 and H929 cells were kind gifts from Dr. Siegfried Janz (University of Iowa, Iowa City, IA, USA) and mouse 5TMM3VT cells were donated by Dr. Wen Zhou (Xiangya School of Medicine, Central South University, Key Laboratory of Carcinogenesis and Cancer Invasion, Ministry of Education; Key Laboratory of Carcinogenesis, National Health and Family Planning Commission, Changsha, China). All the cells were cultured in RPMI-1640 medium (Biological Industries, Beit Haemek, Israel) supplemented with 10% heat-inactivated fetal bovine serum (FBS; Biological Industries, Israel) and 1% penicillin/streptomycin at 37°C with 5% CO_2_. Primary human CD138^+^ cells were collected from the blood samples of each participant and cultured in the same conditions as described above, which was approved by the ethics committees of Affiliated Hospital of Nanjing University of Chinese Medicine (No. 2018NL-KS13).

### Cell Proliferation Assay

Cell growth was evaluated using MTT assay according to the method described in the literature (25). Cells were seeded at a density of 8 × 10^3^ cells/well in 96-well plates. MM cells were cultured with different treatments for 48 h, and primary human CD138+ cells were treated for 24 h at 37°C with 5% CO_2_. 3-(4,5-dimethylthiazol-2-yl)-2,5-diphenyltetrazolium bromide (MTT; 5 mg/mL) was added into each well. After incubation for 4 h, the supernatant was removed and DMSO was added to dissolve formazan. The absorbance was measured at A570 nm with a microplate plate reader (Thermo Fisher Scientific, Inc., USA).

### Apoptosis Assay

MM cells were seeded in 6-well plates with 2 × 10^6^ cells each well and treated with ART (Aladdin, Shanghai, China, #A110206), DHA (MACKLIN, Shanghai, China, #D831931), Dexa (Sigma-Aldrich, Merck KGaA, #D4902) or combination of DHA with Dexa for 48 h. Cells were washed twice with cold prechilled phosphate buffered saline (PBS), resuspended in binding buffer [10 mmol/L HEPES/NaOH (pH 7.4), 140 mmol/L NaCl, and 2.5 mmol/L CaCl_2_], and then stained with annexin-V-FITC (20 μg/mL) and Propidium Iodide (PI; 50 μg/mL; Sigma-Aldrich, Merck KGaA) for 15 min in the dark. The number of apoptotic cells was analyzed using flow cytometer (EMD Millipore; USA; guavaSoft 3.1.1).

### Cell Cycle Analysis

Cells were collected into centrifuge tubes and washed twice with PBS, then fixed with 75% ethanol for 12 h. Subsequently, cells were washed with PBS, treated with 200 μg/mL RNase for 15 min at 37°C to remove RNA contamination, and stained with 50 μg/mL PI (Sigma-Aldrich, Merck KGaA) for 30 min at room temperature in the dark. Cells were filtered through 35 μm mesh to remove clumps before analyzed by a flow cytometer.

### Determination of Δψ_m_

Variations in mitochondrial membrane potential (Δψm) of MM cells were measured using a JC-1 (5,5′,6,6′-tetrachloro-1,1′,3,3′-tetraethylbenzymidazolyl carbocyanine iodide) kit (Beyotime Institute of Biotechnology, Jiangsu, China, #C2006). After treated with 30 μM DHA or 60 μM Dexa for 48 h, a total of 2 × 10^6^ cells were harvested and incubated with JC-1 at 37°C for 20 min and then washed and re-suspended in PBS. The samples were analyzed and 10,000 events were acquired with flow cytometer. JC-1 is presented as monomers in low Δψm (Ex = 549 nm, Em = 590 nm), while it was presented as protomeric aggregates in high Δψm (Ex = 488 nm, Em = 530 nm). The loss of Δψm was rejected by increased green fluorescence from the JC-1 monomers (26).

### Western Blot

Protein levels in MM cells were detected by western blot method, which was performed as previously described (27). MM cells were harvested, washed and lysed with assistance of RIPA Lysis Buffer. 40 μg total protein samples were heated in SDS/β-mercaptoethanol buffer and loaded on 12–15% SDS-PAGE gels. Proteins were separated by electrophoresis in the gels, and then transferred onto PVDF membrane. The membrane was blocked with 5% non-fat milk and incubated with primary antibodies against Bcl-2 (Transduction laboratones, #610538, 1:1,000 dilution), Bad (Cell Signaling Technology, #9292, 1:1,000 dilution), PARP (Cell Signaling Technology, #9542S, dilution rate1:1000), Caspase 3 (Cell Signaling Technology, #9662s, 1:1,000 dilution), cytochrome C (Cyt C; Cell Signaling Technology, #4272, 1:1,000 dilution), and β-actin(Cell Signaling Technology, #4970S, 1:1,000 dilution) overnight at 4°C. Blots were incubated with secondary antibodies used horseradish peroxidase conjugated rabbit anti-mouse (Affinity, #S0002, 1:10,000 dilution) or goat anti-rabbit IgG (Fcmacs, #FMS-Rb01, 1:10,000 dilution) for 1 h at room temperature. Finally, blots were developed by chemiluminescence using ECL kit (Tanon, Shanghai, China).

### Mitochondrial Isolation Assay

Mitochondria isolation was operated according to the manufacturer's instruction of Cell Mitochondria Isolation Kit (Beyotime Institute of Biotechnology, Jiangsu, China, #C3601). MM.1S and MM.1R cells were plated in a 100 mm^2^ dish containing 5 × 10^8^ cells and treated with DHA, Dexa or combination of DHA with Dexa for 48 h. After treatment, cells were washed with cold PBS for three times, then collected into centrifuge tubes (1,200 g for 5 min) and resuspended with isolation buffer containing protease inhibitor (1:1,000, Biolegend, America, CAT: 640941). After standing the suspension for 15 min, the suspension of cells were homogenized by a Dounce glass homogenizer for several complete up-and-down cycles and kept on ice. Next, the liquid was transferred into centrifuge tubes to remove debris including nuclei by centrifugation at 600 g for 10 min at 4°C. Then supernatant was transferred into another centrifuge tube and centrifuged at 11,000 g for 10 min at 4°C. The pellet was the crude mitochondrial fraction. The crude mitochondrial fraction was lysed by mitochondrial lysis fluid for western blot analysis. After placed on ice for 15 min, the lysate was centrifuged at 12,000 g for 5 min at 4°C.

### Oxygen Consumption Rate (OCR) Measurement

O2k (Oroboros, Austria) was used for the measurement of OCR. According to the manufacturer's protocol (https://wiki.oroboros.at/index.php/OROBOROS_INSTRUMENTS), 1 × 10^6^/2 mL ARP1 and H929 cells were cultured for 48 h with 10 μM ART, 10 μM DHA or untreated, respectively. The cell culture medium was replaced with 1,640 medium without FBS, and OCR measurement was performed in real time using software (DatLab 7.0.0.1077, included in the O2k-FluoRespirometer). The mitochondrial inhibitors, 2.5 μM oligomycin (Aladdin, China, #o111756), 1.0 μM carbonyl cyanide 3-chlorophenylhydrazone (FCCP, ACROS, America, #228131000), 0.5 μM rotenone (Aladdin, China, #R105076) and 2.5 μM antimycin A (ENZO, America, #alx-380-075-m005) were added in the listed order (28). The OCR was calculated by the formula:(the max of oxygen consumption after FCCP stimulation—the basal oxygen consumption rate) / protein concentration.

### Measurement of Fe^2+^ Levels

1 × 10^6^ cells were harvested and incubated with 5 μM of Calcein-AM (a non-fluorescent lipophilic ester, Yeasen, Shanghai, China, #40719ES50) for 15 min at 37°C and 50 μg/mL PI for 10 min at 4°C after treated with 10 μM ART or DHA for 48 h. Cells were then washed twice using PBS and the fluorescence intensity signals of the cells were analyzed using a flow cytometry (wavelength of Ex = 490 nm / Em = 515 nm for Calcein-AM; wavelength of Ex = 535 nm / Em = 617 nm for PI). Cytosolic esterases hydrolyze Calcein-AM to release the fluorescent calcein, which binds to the intracellular labile iron pool, resulting in quenching of the fluorescent signal. The cytosolic iron mobilization was calculated by the mean cellular calcein fluorescent intensity using flow cytometry. The reduction of calcein-AM fluorescence intensity represented an increase of chelatable cytosolic Fe^2+^ (29).

### ROS Determination

Intracellular ROS levels were quantified by measuring the fluorescence intensity of the 2′,7′-dichlorofluorescein diacetate (DCFH-DA; Beyotime Institute of Biotechnology, Jiangsu, China, #S0033) with PI as a probe using flow cytometry. The non-fluorescent DCFH-DA is deacetylated by intracellular esterases to the non-fluorescent DCFH, which is subsequently rapidly oxidized by intracellular ROS to the fluorescent 2′,7′-dichlorofluorescein. Cells were washed twice with PBS and incubated with 10 μM DCFH-DA at 37°C for 30 min in the dark. Subsequently, the cells were washed twice and resuspended in PBS. ROS levels were determined using a flow cytometer.

### Human Myeloma Xenograft Mice Model

All animal procedures were conducted in accordance with government-published recommendations for the Care and Use of Laboratory Animal, which were approved by the Institutional Ethics Review Boards of Nanjing University of Chinese Medicine (No. ACU170501 and 201905A003). MM.1S (sensitive to Dexa) and MM.1R (resistant to Dexa) cells (3 × 10^6^) were subcutaneously injected into the abdominal area of 6~8-weeks old NOD-SCID mice (*n* = 10 per group) from Beijing Vital River Laboratory Animal Technology, Co., Ltd (Beijing, China). Starting on day 3 post cell transfer, mice were treated with DHA (25 mg/kg) three times a week and Dexa (9 mg/kg) every other day. The tumor volumes were measured using calipers at the indicated time points. When the tumor diameters reached 20 mm, the mice were sacrificed. Tumor volume (mm^3^) was calculated as: (length × width^2^)/2 (30).

### 5TMM3VT Myeloma Mice Model

5TMM3VT murine myeloma cells (1 × 10^6^) were injected through the tail vein into 6-week-old C57BL/KaLwRij mice (*n* = 10 per group). The mice were divided into 3 groups as follows: DHA (50 mg/kg) treatment group, ART (50 mg/kg) group, and control group (Castor oil: ethanol: saline=2:1:7). After 2 days, 10 mice from each group were treated via intraperitoneal injection three times a week for 75 days until all the mice were dead. DHA and ART were dissolved in 70% saline, 20% Castor oil, and 10% ethanol.

### Statistical Analysis

Data were expressed as the mean ± SD. The Student's *t*-test was used to determine a significant difference. The difference between groups was set at ^*^p < 0.05, ^**^p < 0.01, and ^***^p < 0.001. Mouse survival was analyzed by GraphPad Prism 5 software (GraphPad Software Inc., La Jolla, CA, USA) using the Log-rank (Mantel-Cox) Test. The interaction between DHA and Dexa was analyzed by CalcuSyn software program (CalcuSyn Version 2.1, Biosoft). Isobologram analysis was based on the Chou-Talalay method with the combination index (CI). CI <1.0 indicates synergism, CI = 1.0 presents additive activity and CI > 1.0 states antagonism.

## Results

### DHA Is a Potential Drug in the Treatment of Myeloma

**Figure 1:**
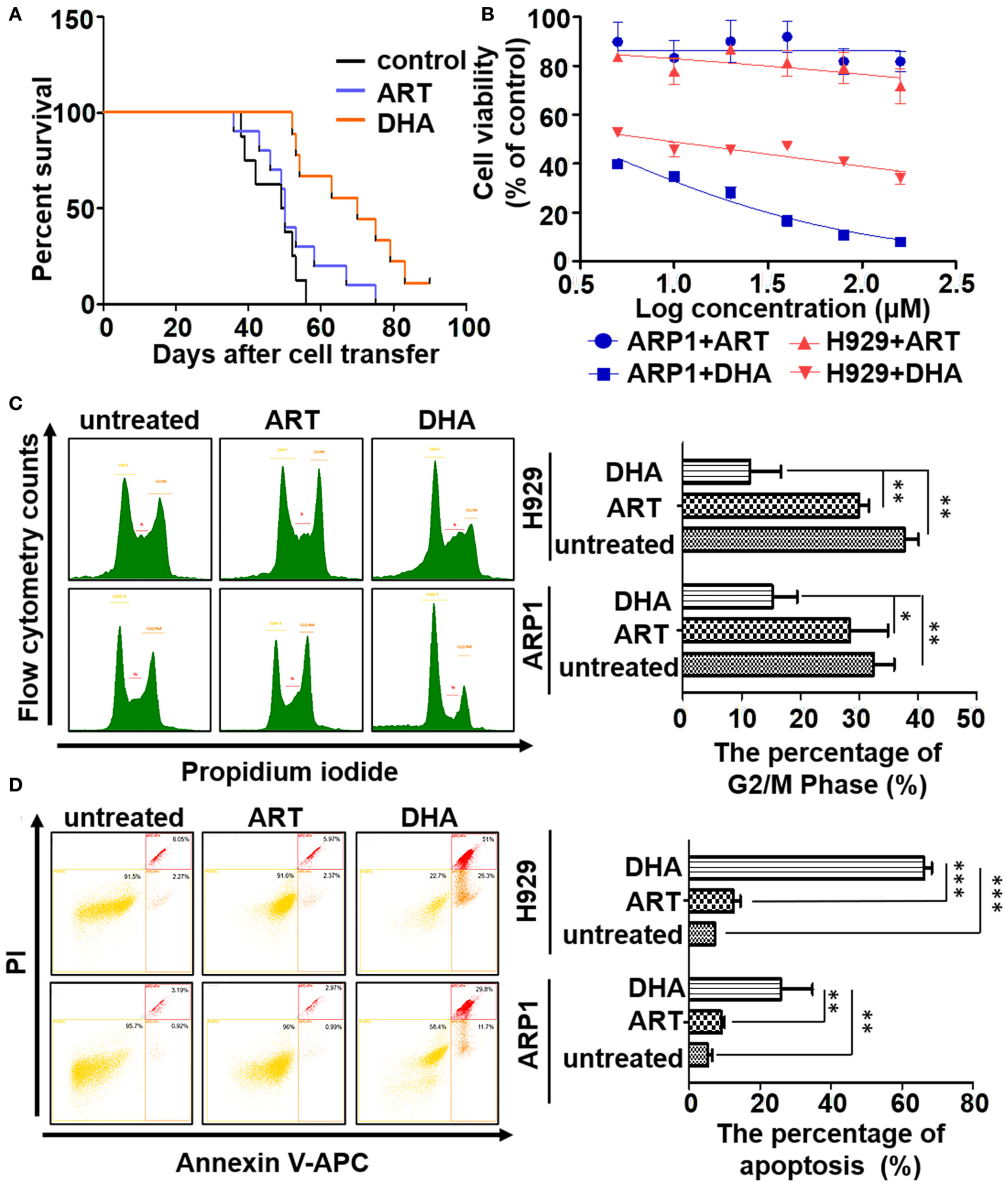
DHA is a potential drug in the treatment of myeloma. **(A)** The survival data was obtained using 5TMM3VT myeloma mice model. **(B)** The MTT assay. DHA could suppress the proliferation of ARP1 and H929 cells, while ART could not. **(C,D)** The cell cycle and apoptosis of MM cells with or without treatment of either DHA or ART were determined by flow cytometry. The difference of the cell cycle of G2/M phase or apoptosis between groups was calculated by Student's *t*-test. *P < 0.05, **P < 0.01, and ***P < 0.001 were considered statistically significant.

To evaluate the potential of ART or DHA as a treatment for MM, the therapeutic effects of ART and DHA were determined on overall survival rate of C57BL/KaLwRij MM-prone mouse model established using 5TMM3VT cells. Kaplan–Meier survival curve showed that the MM mice with ART treatment had significantly improved overall survival (median survival, 53 days) compared with the untreated control animals (median survival, 38 days; *P* = 0.0085). Additionally, MM mice treated with DHA had a significantly longer survival (median survival >75 days) compared with the untreated control animals (*P* = 0.0020). None of the untreated control mice survived >6 weeks; however, mice treated with ART survived for <8 weeks (39% increase) while mice treated with DHA survived >10 weeks (90% increase). The improved survival rate of DHA-treated mice compared with those of ART-treated mice (*P* = 0.0116; Figure 1A) demonstrated the therapeutic potential of DHA compared with ART for the treatment of MM.

The effects of ART and DHA on the proliferation of the MM cell lines were determined (Figure 1B). Treatment of ARP1 and H929 cells with ART or DHA resulted in dose-dependent cytotoxicity. The IC_50_ of ART was significantly higher than that of DHA in both ARP1 (2.84 mM vs. 2.937 μM, respectively) and H929 (815 μM vs. 7.931 μM) MM cells (Figure 1B), highlighting the better efficacy of DHA in the treatment for MM. This observation was further confirmed by performing a cell cycle assay and apoptosis analysis. In the cell cycle assay, the proportion of ART- and DHA-treated cells in the G2/M stages decreased compared with that in the untreated control group, and the proportion of ARP1 (15.23 ± 3.66%) and H929 cells (11.46 ± 4.26%) in these phases was significantly lower in the DHA-treated group than that in the ART-treated group (ARP1 cells: 28.40 ± 5.63%, *P* < 0.05 and (H929 cells: 29.97 ± 1.38%, *P* < 0.01; Figure 1C). Flow cytometry analysis of apoptosis showed that there was a significant increase in the apoptotic levels in the DHA-treated ARP1 (25.83 ± 7.80%) and H929 cells (66.23 ± 1.80%) compared with the ART-treated ARP1 (9.08 ± 0.72%, *P* < 0.01) and H929 cells (12.54 ± 1.59%, *P* < 0.01) and the untreated ARP1 (5.35 ± 0.96%, *P* < 0.001) and H929 cells (7.29 ± 0.14%, *P* < 0.001; Figure 1D).

### DHA Treatment Increases the Concentration of ROS in MM Cell Lines

**Figure 2:**
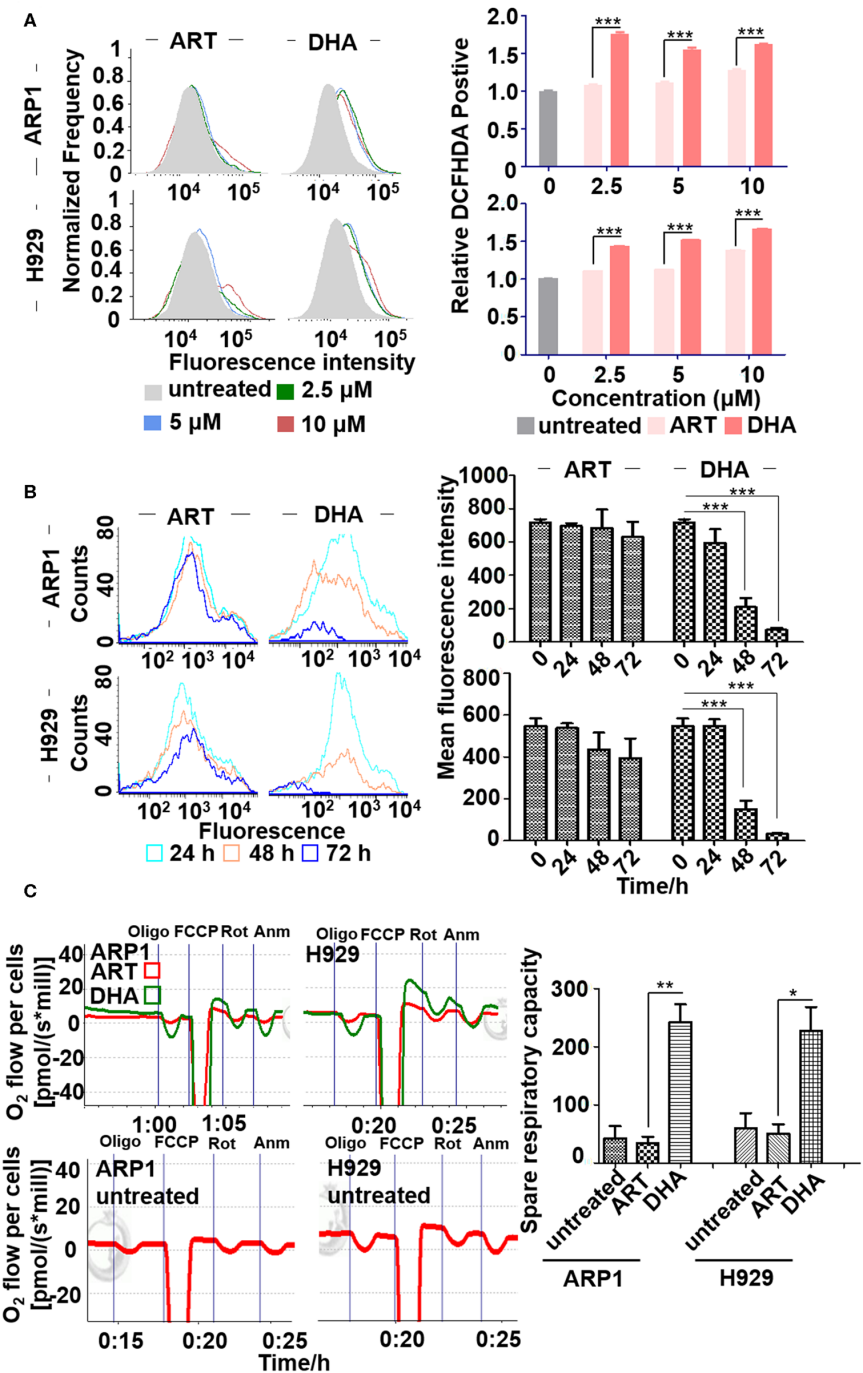
DHA increases the concentration of reactive oxygen species (ROS) in MM cell lines. **(A)** ROS determination assay. ARP1 and H929 cells were treated with different concentrations of ART or DHA for 48 h, and the percentage of DCFHDA positive cells was detected by flow cytometry. **(B)** The measurement of Fe^2+^ levels. Mean fluorescence intensity of ARP1 and H929 cells treated with ART or DHA for different time points (24, 48, and 72 h) was measured by flow cytometry. **(C)** The measurement of OCR. The oxygen consumption rate was determined by O2k (see Materials and Methods). Data statistics were analyzed by Student's *t*-test. *P < 0.05, **P < 0.01, ***P < 0.001.

ROS serve an important role during apoptosis (31) and ROS concentration is the most intuitive indicator to evaluate the oxidant activity of ART and DHA (32). Thus, the ROS levels in MM cells treated with ART or DHA were determined. As shown in Figure 2A, DHA significantly increased ROS production compared with ART treatment (*P* < 0.001) in ARP1 and H929 cells. At 2.5 μM DHA in ARP1 cells, the generation of ROS (1.65 ± 0.06) was significantly higher than that in the untreated cells (1.00 ± 0.02) and ART-treated cells (1.15 ± 0.10). Similar results were observed in H929 cells and at other concentrations, suggesting that DHA increased intracellular ROS production compared with the ART and untreated cells (Figure 2A).

Previous studies have reported that ART treatments, in the presence of free iron, result in the production of alkylating agents which damage cancer cells (33). Therefore, the effect of DHA on intracellular iron in MM cells was examined. The fluorescence intensity of intracellular iron staining was determined using Calcein-AM after 0, 24, 48 and 72 h of treatment and flow cytometry (Figure 2B). The cytoplasmic Fe^2+^ of MM cells was labeled using Calcein-AM and an increase in fluorescence intensity represented reduced ferrous iron content. The fluorescence intensity of calcein was lower in ARP1 (0 vs. 48 h, 715.61 ± 21.72 vs. 209.46 ± 54.75) and in H929 cells (0 vs. 48 h, 547.73 ± 36.4 vs 149.07 ± 41.34) treated with DHA for 48 h. Calcein fluorescence intensity was significantly lower in cells treated with DHA after 48 h than in ART-treated and untreated cells.

To determine whether ART and DHA altered mitochondrial OCR, MM cells were treated with ART or DHA for 48 h. Oligomycin A was added to the cells to examine oxygen consumption coupled with ATP synthesis. The uncoupler carbonyl cyanide-4-(trifluoromethoxy) phenylhydrazone (FCCP) was added to determine the maximal respiratory capacity. Antimycin A and rotenone were used to determine the spare respiratory capacity. As shown in Figure 2C, only treatment with DHA resulted in a significant increase in OCR, suggesting that DHA, but not ART, increases the concentration of ROS primarily through increasing mitochondrial OCR.

### DHA Induces the Mitochondrial Apoptosis Pathway in MM

**Figure 3:**
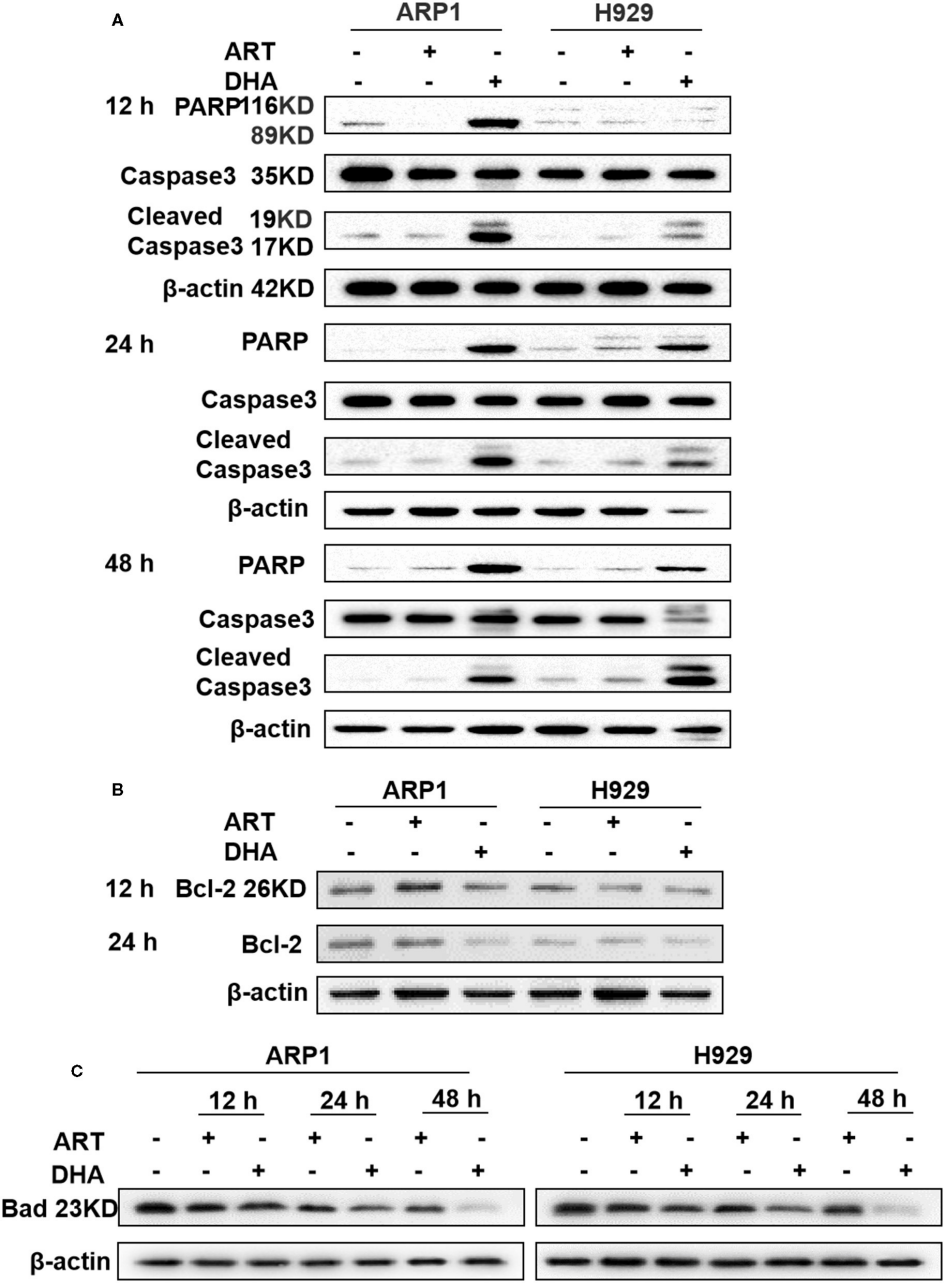
DHA induces caspase-mediated apoptosis in MM cell lines. **(A–C)** The protein levels of caspase-3, PARP, Bcl-2, and Bad were determined by western blotting in MM cells. The results showed that caspase-3 and PARP were increased after 12 h, while Bcl-2 and Bad were decreased after 12 h.

To confirm the effect of DHA on MM cell apoptosis and the involvement of the mitochondrial pathway, the expression of caspase-3, PARP, Bcl-2, and Bad were determined, which were indicators of apoptosis in a number of different types of cancer (34). Western blot showed that cleaved caspase-3 and PARP expression levels in ARP1 and H929 cells treated with DHA were significantly increased compared with cells treated with ART or untreated cells after 12, 24, and 48 h (Figure 3A). Bcl-2 expression levels were significantly decreased in cells treated with DHA after 24 h (Figure 3B). To further confirm the involvement of mitochondria-dependent apoptosis in cells treated with DHA, Bad expression was measured. As shown in Figure 3C, DHA treatment downregulated the expression of Bad in both ARP1 and H929 cells in a time-dependent manner.

### DHA Overcomes Resistance to Dexa in MM

**Figure 4:**
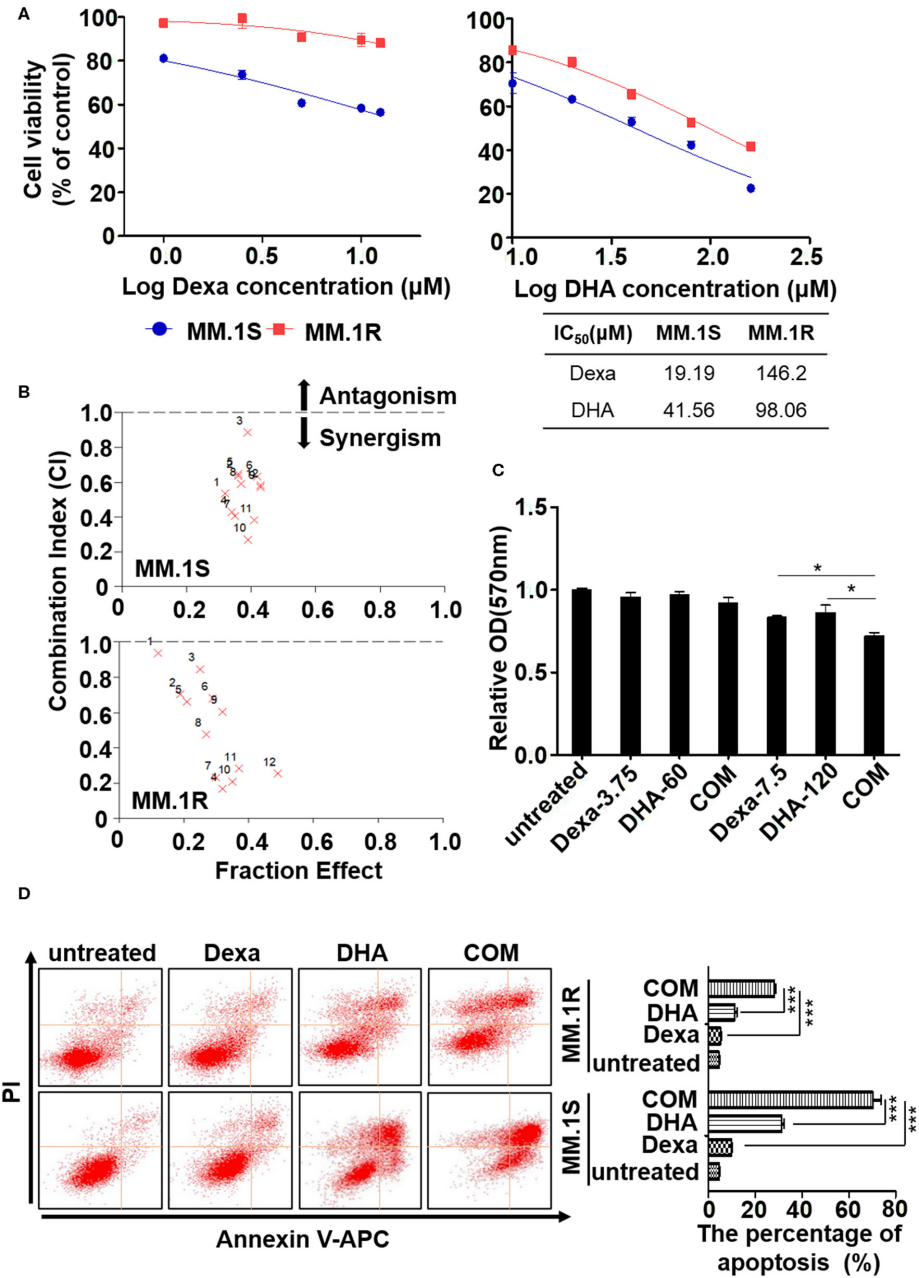
DHA overcomes dexamethasone resistance in multiple myeloma. **(A)** The MTT assay. DHA suppresses the proliferation of ARP1 and H929 cells. **(B)** DHA synergistically enhances the effect of Dexa. **(C)** The MTT assay. The combination of DHA and Dexa significantly inhibits the growth of CD138 positive cells. **(D)** The apoptosis of MM.1S and MM.1R cells was measured by staining with Annexin V and PI, which was followed by flow cytometry analysis. *P < 0.05, ***P < 0.001.

Since Bcl-2 is a typical marker for Dexa resistance MM patients, we further assessed the efficacy of DHA for treatment of Dexa-resistant MM (35). Paired Dexa-sensitive (MM.1S) and Dexa-resistant (MM.1R) MM cell lines were utilized, which were established from peripheral blood of the same MM patient and sensitive/resistant to Dexa, respectively (36). The viability of MM.1S and MM.1R cells significantly reduced in a concentration-dependent manner (Figure 4A). Of note, the MM.1R cells exhibited some sensitivity to DHA.

As Dexa is the most widely used treatment for patients with MM despite the development of drug resistance (37), the effect of DHA in combination with Dexa was determined. As shown in Figure 4B, a combination of DHA and Dexa significantly reduced the viability of MM.1S (combination index, CI, of 17.5 μM DHA with 60 μM Dexa, 0.269) and MM.1R cells (CI of 17.5 μM DHA with 15 μM Dexa, 0.167). Comparable results of combined treatment and Dexa were observed, confirming that combined treatment significantly reduced viability compared with either treatment alone, irrespective of the Dexa dose.

To determine the clinical potential of DHA combined with Dexa, MM cells obtained from the MM patients were treated with the drugs to evaluate their effects on growth in short-term cultures for 12 h. The CD138^+^ cells showed significantly reduced growth when treated with the combination of drugs compared with 7.5 μM Dexa or 120 μM DHA alone (both *P* < 0.05; Figure 4C). Additionally, apoptosis was detected using flow cytometry, and it was demonstrated that both MM.1S and MM.1R cells treated with the combination of drugs exhibited increased apoptosis compared with Dexa or DHA alone (both *P* < 0.001; Figure 4D), consistent with the results of ARP1 and H929 cells treated with the drugs either alone or in combination.

### DHA Combined With Dexa Increases Translocation of Cytochrome C (cyt C) From the Mitochondria to the Cytoplasm

**Figure 5:**
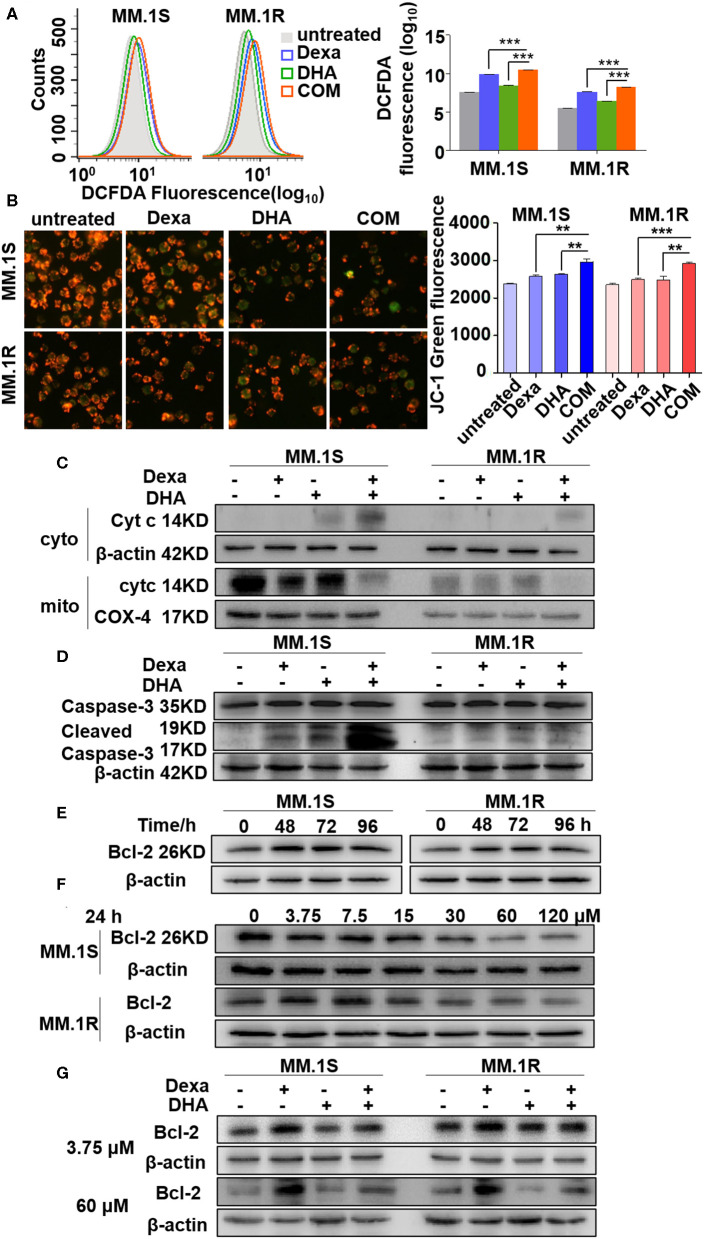
DHA in combination with Dexa induced more translocation of Cyt C from the mitochondria to the cytoplasm. **(A,B)** Determination of ROS and mitochondrial membrane potential. Fluorescence intensity is detected by flow cytometry. **(C–G)** The western blotting results showed that Cyt C and cleaved caspase-3 are increased after treatment with DHA plus Dexa and Bcl-2 expression is associated with Dexa administration in a time- and concentration-dependent manner. **P < 0.01, ***P < 0.001.

In order to detect the mechanism through which DHA overcomes Dexa resistance, we further explored if DHA combined with Dexa could increase oxidative stress and ROS production in MM.1S and MM.1R cells. As shown in Figure 5A, the levels of ROS in cells treated with a combination of drugs were significantly higher than those in cells treated with either treatment alone. Treatment with Dexa or DHA alone, or combined resulted in a loss in Δψ_m_, which is represented by an increase in JC-1 green fluorescence (Figure 5B). The combination treatment (2965.06 ± 64.52 in MM.1S cells; 2918.14 ± 26.21 in MM.1R cells) was significantly more effective compared with Dexa alone (2579.85 ± 36.75 in MM.1S cells, *P* < 0.01; 2492.58 ± 36.39 in MM.1R cells, *P* < 0.01) and DHA alone (2632.60 ± 8.91 in MM.1S cells, *P* < 0.001; 2477.90 ± 84.07 in MM.1R cells, *P* < 0.01).

The alterations of Δψ_m_, induced by a combination of drugs, are associated with translocation of mitochondrial proteins to the cytosol, including Cyt C (38). As shown in Figure 5C, treatment with the combination of drugs resulted in increased translocation of Cyt C to the cytosol compared with treatment with either Dexa or DHA alone. It has been reported that during mitochondrial permeability transition, Cyt C activates caspase-3 (39). The expression levels of cleaved caspase-3 were increased by DHA and Dexa alone, and further increased when cells were treated with a combination of the two drugs (Figure 5D). Cyt Ctranslocation from the mitochondria to the cytosol depends partly on the expression of Bcl-2 (40), thus Bcl-2 expression in cells treated with Dexa, DHA, or both combined was examined. Additionally, Dexa increased Bcl-2 expression in a time-dependent manner (0–96 h) peaking at 48 and 72 h with the concentration of 3.75 μM, in MM.1S and MM.1R cells (Figure 5E), suggesting that Dexa may increase Bcl-2 expression during the first 24 h. However, increasing the concentration of Dexa (0, 3.75, 7.5, 15, 30, 60, and 120 μM) within 24 h resulted in a decrease in Bcl-2 expression including in the MM.1R cells (Figure 5F). Irrespective of the concentration of Dexa used, the combination treatment restored the Dexa-induced Bcl-2 expression in the resistant cells, suggesting that DHA treatment was able to overcome Dexa resistance.

These results show that the cytotoxic effects of DHA combined with Dexa were mediated by increased ROS production, altering Δψ_m_, reversing changes to Bcl-2 expression mediated by Dexa, increasing release of mitochondrial Cyt C, activating caspase-3, and finally inducing caspase-mediated apoptosis.

### Anti-MM Activity of DHA Combined With Dexa in a MM Xenograft Mouse Model

**Figure 6:**
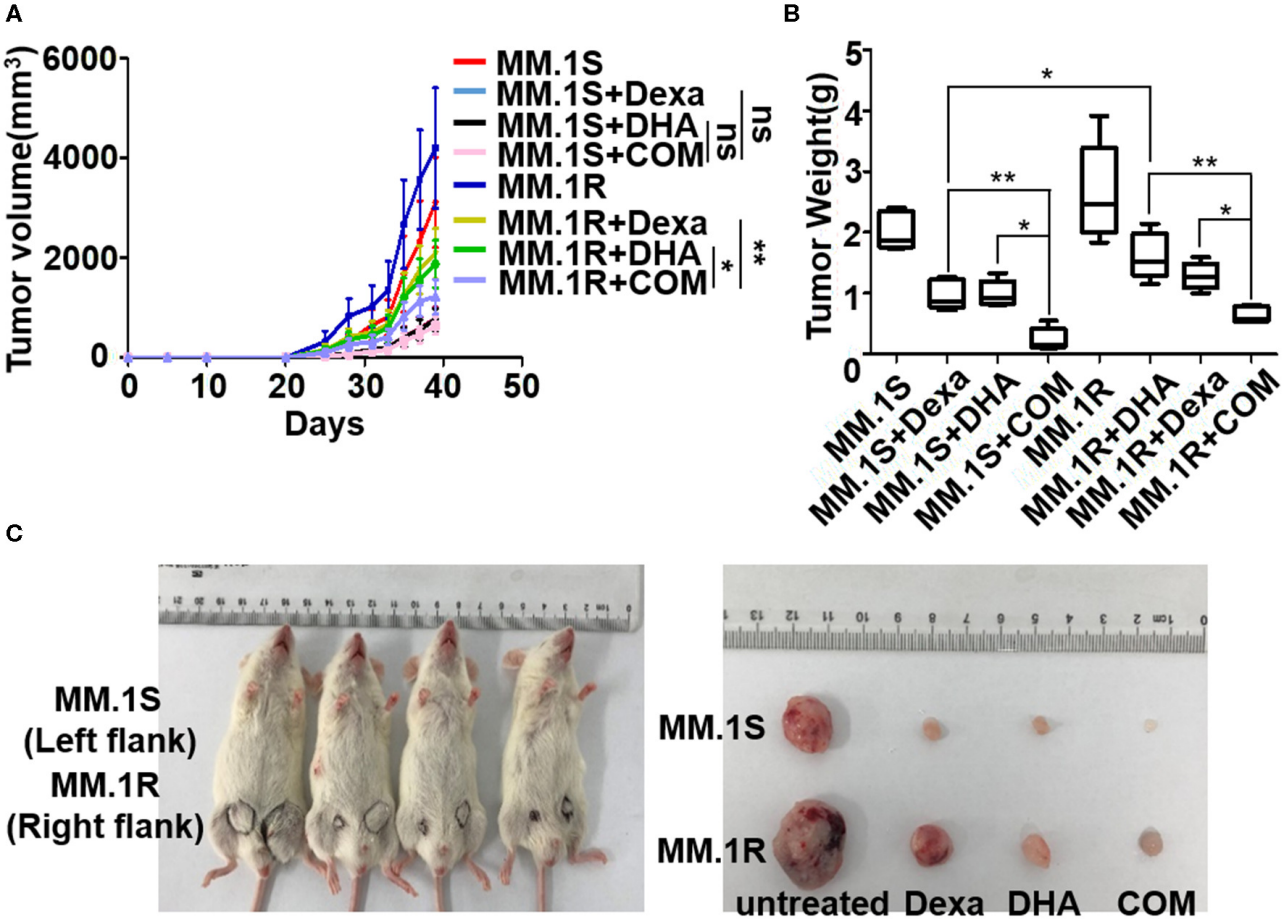
Anti-MM activity of DHA in combination with Dexa in MM xenograft mouse models. **(A)** The effect of DHA combined with Dexa (COM) was examined in subcutaneous transplantation mouse models using MM.1S and MM.1R cell lines. The result indicated that COM significantly inhibited MM.1R tumor growth compared with the DHA and ART group. **(B)** After treatment, the tumor weight of COM group was lower than that of the DHA or ART group. *P < 0.05, **P < 0.01. **(C)** The NOD-SCID mice injected subcutaneously with MM.1S (left) and MM.1R (right) cells were sacrificed and tumors were isolated on Day 39.

To replicate our findings *in vivo*, an MM xenograft mouse model was used to evaluate the antitumor activity of DHA in combination with Dexa. NOD-SCID mice were injected with MM.1S or MM.1R cells subcutaneously; tumor neoplasms in untreated mice developed over ~1 month, whereas tumor growth of the Dexa- or DHA-treated mice developed slower compared with the untreated mice (Figure 6A). The combination treatment resulted in significantly reduced tumor growth compared with either treatment alone in mice injected with MM.1R cells (COM 1,217.76 ± 343.15 mm^3^ vs. Dexa 2079.50 ± 507.87 mm^3^, *P* < 0.001; COM vs. DHA 1875.53 ± 475.61 mm^3^, *P* < 0.05). However, this was not observed in mice injected with MM.1S cells (Figure 6A). After 39 days, the weight of tumors from the mice treated with both drugs (0.24 ± 0.17 g in MM.1S; 0.65 ± 0.12 g in MM.1R cells) was significantly reduced compared with that noted for untreated mice (2.02 ± 0.27 g in MM.1S; 2.65 ± 0.72 g in MM.1R), Dexa treated mice (0.98 ± 0.22 g in MM.1S; 1.62 ± 0.34 g in MM.1R), and DHA-treated mice (0.99 ± 0.19 g in MM.1S; 1.28 ± 0.20 g in MM.1R) (Figures 6B,C).

## Discussion

Despite the advancements in therapeutic target discovery and targeted therapy, MM is still incurable. Typically, MM patients respond to the treatment initially, but the majority of them ultimately relapse. Therefore, the development of novel treatment is extremely urgent. Artemisinin-type drugs are typically metabolized *in vivo* to the active metabolite DHA (41), and the anticancer effects of ART and its derivative DHA have been demonstrated in a number of different types of cancer (41–45). For instance, in breast cancer, DHA induced apoptosis and G0/G1 arrest by activating Bid, increased the expression of Bim, decreased the expression of Bcl-2, induced the translocation of Cyt C from the mitochondria to the cytosol, and increased the expression of caspase-8, and cleaved caspase-9 (43, 44). However, so far, there have been no studies examining the effects of DHA on MM, and in particular, the combination of DHA with other chemotherapeutic drugs including Dexa on MM. Our study first evaluated the effects of ART and DHA on MM *in vitro* and *in vivo*, and demonstrated that DHA could possess a greater efficacy than ART against MM (Figures 1A,B). At the cellular level, ART could only induce weak apoptosis of MM cells and decrease the G2/M staged cells (Figures 1C,D) compared to DHA, suggesting that ART could be converted into activated DHA in the microenvironment of MM cells. The *in vivo* study in 5TMM3VT myeloma C57BL/KaLwRij mice showed that both ART and DHA could improve the survival rate of the mice; however, MM mice treated with DHA had a longer survival than ART-treated mice. This phenomenon may be due to the fact that ART *in vivo* is not completely converted to the activated DHA.

**Figure 7:**
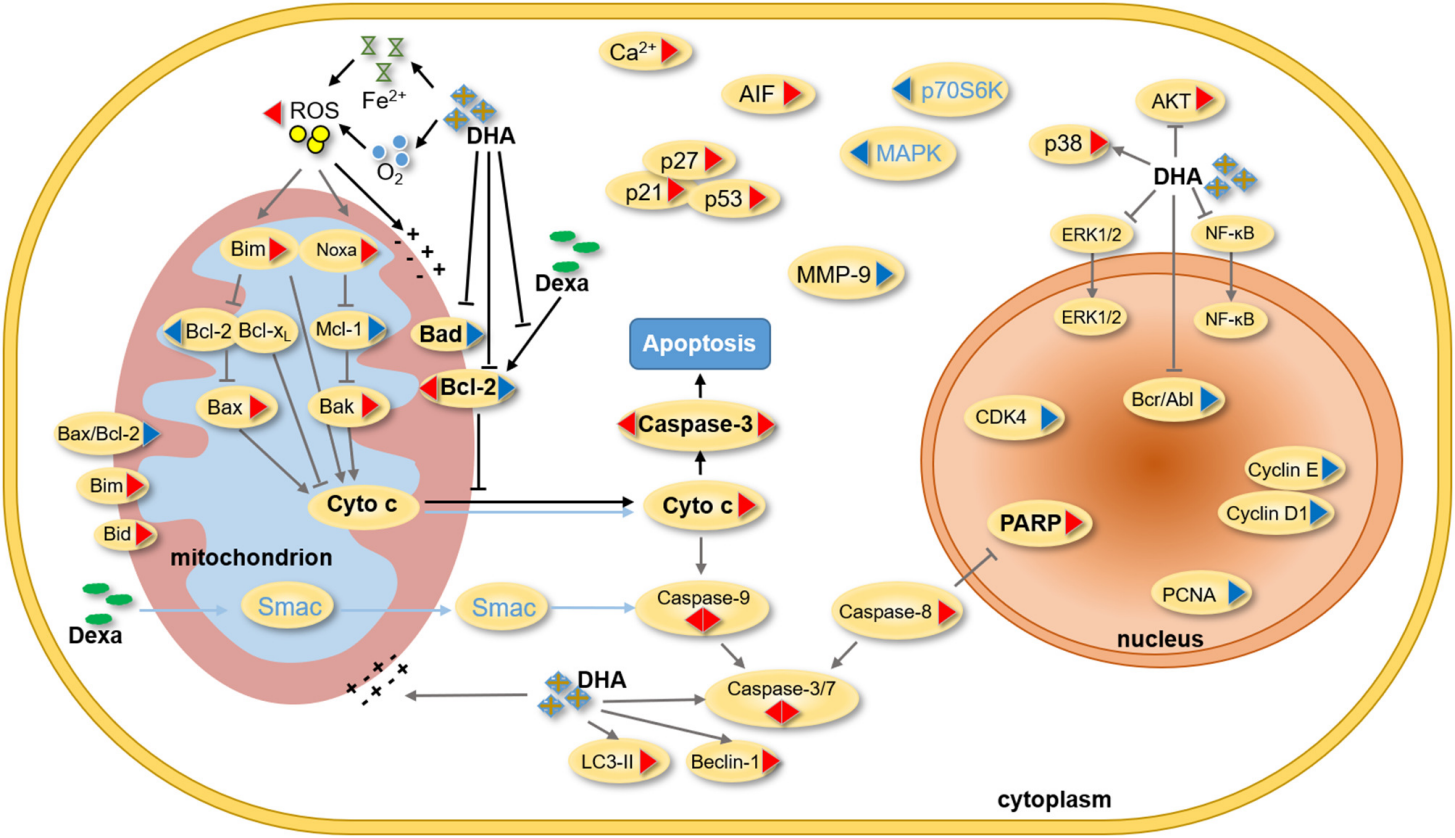
Summary of the effect of DHA and Dexa on MM. In previous studies, DHA was found to influence the progression of different cancers by regulating the expression of mitochondrial, nucleus and cytoplasmic proteins. On protein level, Dexa inhibits the proliferation of MM cells by mainly adjusting the expression of Smac, cytochrome C, caspase-3, caspase-9, MAPK and p70S6K. In our study, DHA was found to not only induce the mitochondrial apoptosis pathway, but also inhibit Bcl-2 expression induced by Dexa. A synergism of DHA and Dexa increases ROS levels and translocation of cytochrome C from mitochondria to the cytoplasm, and then alters mitochondrial membrane potential as well as caspase-mediated apoptosis. (The triangle on the left shows the effect of Dexa on the protein expression, and the other side shows the effect of DHA on the protein expression. The red triangle indicates an increase in protein expression and the blue triangle indicates a decrease in the protein expression. The blue characters and lines represent Dexa-acting proteins. The black line and the bold characters represent the summary of this study, and the gray line represents the summary of the literature study).

Consistent with previous reports that DHA prevented MM tumor growth through a caspase-mediated pathway (43), the current study found that the mitochondria were the primary target by which DHA exerted its effects. DHA activated apoptosis via caspase-3, followed by PARP cleavage (Figure 3A), and decreased Δψ_m_ (Figure 5B), which in turn, increased the translocation of Cyt C from the mitochondria to the cytoplasm (Figure 5C) (45). Additionally, DHA interacted with iron and activated oxygen consumption (Figures 2B,C), resulting in increased production of ROS (Figures 2A, 5A), and this was assumed to be the after-effects of permeabilization of the outer mitochondrial membrane (46), since outer mitochondrial membrane permeabilization resulted in a loss of Δψ_m_ and an increase in ROS levels (47). For MM treatment, the drug resistance and its related relapse are the major clinical obstacles, we further explored that DHA is effective in treating drug-resistant MM (Figures 4A,B). DHA-induced mitochondrial apoptotic pathways are largely related to those pathways affected by Dexa. Dexa increases the releasing of the mitochondrial apoptogenic factors Second mitochondrial-derived activator (Smac)/DIABLO and Cyt C from the mitochondria to the cytosol, and activates the SAPK/JNK-independent pathway, which is associated with downregulation of MAPK and p70S6K (48). However, DHA reduced the expression of Bad and Bcl-2 (Figures 3B,C), while Dexa increased Bcl-2 expression in both MM.1S and MM.1R cells within the first 48 h at lower concentrations (Figure 5E) and suppressed the growth of MM cells at higher concentrations (Figure 5F). Therefore, we infer that DHA might counteract the effects of Dexa to balance Bcl-2 expression (Figure 5G), thus regulating the translocation of Cyt C to further promote cell apoptosis (summarized in Figure 7). This hypothesis is further strengthened by the report that Bcl-2 inhibits the translocation of Cyt C from the mitochondria to the cytoplasm (49), and Cyt C increases caspase-3 expression and ultimately induces caspase-3-mediated apoptosis (50).

Therefore, we further confirmed that DHA could overcome the Dexa resistance in MM (Figures 4A–C). DHA-induced cytotoxicity in MM.1S and MM.1R cells is enhanced by Dexa, suggesting that the apoptotic signaling cascades by which DHA and Dexa exert their effects may be different (Figure 4D). It has been demonstrated that Dexa-induced caspase-9 activation via a Smac-dependent and Cyt C-independent pathway (51), whereas, in the present study, DHA activated caspase-3 and increased the release of Cyt C from the mitochondria (Figures 5C,D). DHA synergistically augmented the effect of Dexa-induced cytotoxicity (Figure 4B), including the decrease in proliferation and the increase in apoptosis of MM cells (Figure 4D). Importantly, DHA re-sensitized MM.1R cells to Dexa *in vitro* and *in vivo* (Figures 6A–C). Through these mechanisms, DHA alone not only exhibits anticancer effects in MM but also augments and complements the effects of the widely used anti-MM agent Dexa. These data provide a rationale for future clinical studies to develop a novel therapeutic regimen comprising DHA alone or in combination with other agents for patients with MM particularly for relapsed/refractory MM patients.

In conclusion, our study provides the first evidence that DHA may be an effective standalone treatment for MM patients and that it can overcome Dexa resistance. Mechanistically, DHA prevents MM development and progression through mitochondrial apoptotic pathway involving Fe^2+^ and oxygen. DHA augments the effects of Dexa, resulting in increased production of ROS and translocation of Cyt C from the mitochondria to the cytoplasm, and downregulates Dexa-induced expression of Bcl-2, a biomarker of Dexa resistance. Therefore, DHA may be a promising therapeutic option for patients with refractory/relapsed MM. Further studies are necessary to determine the clinical efficacy and detailed therapeutic targets.

## Data Availability Statement

The raw data supporting the conclusions of this article will be made available by the authors, without undue reservation, to any qualified researcher.

## Ethics Statement

The studies involving human participants were reviewed and approved by The operations of primary human CD138^+^ cells were approved by the ethics committees of Affiliated Hospital of Nanjing University of Chinese Medicine (No. 2018NL-KS13). The patients/participants provided their written informed consent to participate in this study. The animal study was reviewed and approved by Animals experiments were approved by the Ethics Committee of Nanjing University of Chinese Medicine (Nanjing, China; animal ethics registration no. ACU170501 and 201905A003).

## Author Contributions

This study was designed and conceived by YY and CG. Experiments were performed by YC, RL, YZ, SZ, and JQ. DY performed data analysis. AJ and MB provided technical counseling on experiments. YC wrote the manuscript. YY and CG revised the manuscript. All authors read and approved the final version of the manuscript.

## Conflict of Interest

The authors declare that the research was conducted in the absence of any commercial or financial relationships that could be construed as a potential conflict of interest.
