# Corrigendum: Dihydroartemisinin Induces Growth Arrest and Overcomes Dexamethasone Resistance in Multiple Myeloma

**DOI:** 10.3389/fonc.2021.736373

**Published:** 2021-07-23

**Authors:** Ying Chen, Rui Li, Yuqi Zhu, Sixia Zhong, Jinjun Qian, Dongqing Yang, Artur Jurczyszyn, Meral Beksac, Chunyan Gu, Ye Yang

**Affiliations:** ^1^ The Third Affifiliated Hospital of Nanjing University of Chinese Medicine, Nanjing, China; ^2^ School of Medicine & Holistic Integrative Medicine, Nanjing University of Chinese Medicine, Nanjing, China; ^3^ Department of Internal Medicine, University of Iowa, Iowa City, IA, United States; ^4^ Department of Hematology, Collegium Medicum, Jagiellonian University, Kraków, Poland; ^5^ Department of Hematology, School of Medicine, Ankara University, Ankara, Turkey; ^6^ Key Laboratory for Combination of Acupuncture and Chinese Materia Medica of Chinese Ministry of Education, Nanjing University of Chinese Medicine, Nanjing, China

**Keywords:** dihydroartemisinin, artemisinin, multiple myeloma, dexamethasone, drug resistance

In the original article, there was an unsatisfied WB result in ***Figure 3*** as published. Dr. Tulipa Fosteriana pointed that in ***Figure 3C***, the 2 loading controls bared striking resemblance, although they looked somehow different in vertical dimension and the image quality was also quite poor, which were posted on PubPeer. According to the valuable suggestions, we have provided all the original data to Frontiers Office to prove the authenticity. There was no error in the original data, however, based on the recommendation from Editorial Office, we replaced the previous figure with the repeated “***C of Figure 3***” with high quality. The corrected ***Figure 3*** appears below.

**Figure 3:**
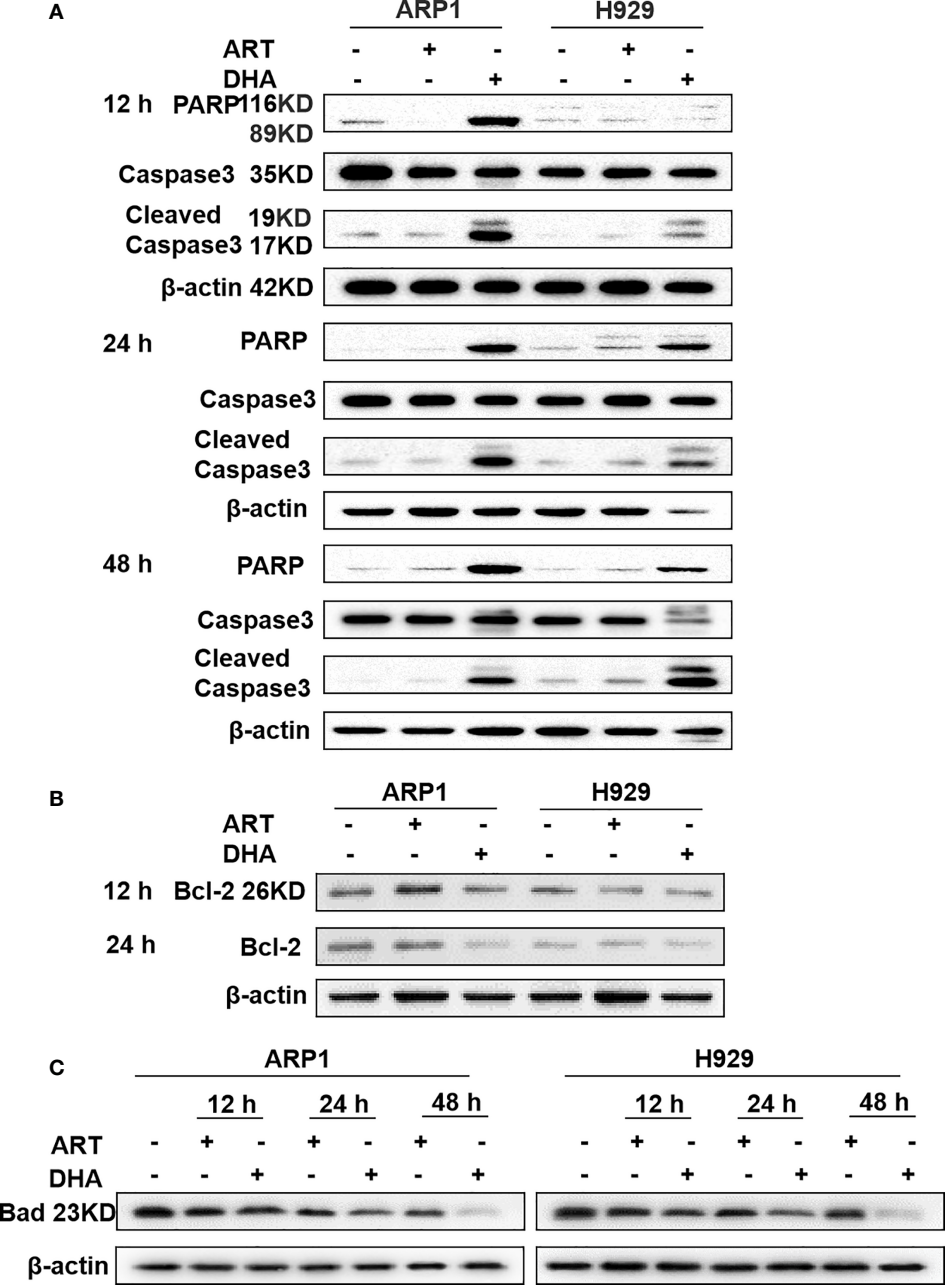
DHA induces caspase-mediated apoptosis in MM cell lines. **(A–C)** The protein levels of caspase-3, PARP, Bcl-2, and Bad were determined by western blotting in MM cells. The results showed that caspase-3 and PARP were increased after 12 h, while Bcl-2 and Bad were decreased after 12 h.

The authors state that this does not change the scientific conclusions of the article in any way. The original article has been updated.

## Publisher’s Note

All claims expressed in this article are solely those of the authors and do not necessarily represent those of their affiliated organizations, or those of the publisher, the editors and the reviewers. Any product that may be evaluated in this article, or claim that may be made by its manufacturer, is not guaranteed or endorsed by the publisher.

